# PKCδ Mediates NF-*κ*B Inflammatory Response and Downregulates SIRT1 Expression in Liver Fibrosis

**DOI:** 10.3390/ijms20184607

**Published:** 2019-09-17

**Authors:** Su Jin Lee, Su Ji Kim, Hyun-Shik Lee, Oh-Shin Kwon

**Affiliations:** School of Life Science and Biotechnology, BK21 Plus KNU Creative BioResearch Group, College of Natural Science, Kyungpook National University, Daegu 41566, Korea; neojove79@naver.com (S.J.L.); susie514@hanmail.net (S.J.K.); leeh@knu.ac.kr (H.-S.L.)

**Keywords:** PKC*δ*, NF-κB, SIRT1, hepatic cirrhosis and LPS

## Abstract

The precise mechanism of hepatic cirrhosis remains largely unclear. In particular, a potential regulatory mechanism by which protein kinase C-delta (PKC*δ* ) affects profibrogenic gene expression involved in hepatic cirrhosis has never been explored. In the present study, we investigated whether PKC*δ* activation is involved in liver inflammatory fibrosis in both lipopolysaccharide (LPS)-treated RAW 264.7 and CCl_4_-treated mice. PKC*δ* was strongly activated by LPS or CCl_4_ treatment and consequently stimulated nuclear factor (NF)-κB inflammatory response. Interestingly, the activation of PKC*δ* negatively regulated sirtuin-1 (SIRT1) expression, whereas PKC*δ* suppression by PKC*δ* peptide inhibitor V1-1 or siRNA dramatically increased SIRT1 expression. Furthermore, we showed that the negative regulation of PKCδ leads to a decrease in SIRT1 expression. To our knowledge, these results are the first demonstration of the involvement of PKC*δ* in modulating NF-κB through SIRT1 signaling in fibrosis in mice, suggesting a novel role of PKC*δ* in inflammatory fibrosis. The level of NF-κB p65 in the nucleus was also negatively regulated by SIRT1 activity. We showed that the inhibition of PKC*δ* promoted SIRT1 expression and decreased p65 levels in the nucleus through deacetylation. Moreover, the inactivation of PKC*δ* with V1-1 dramatically suppressed the inflammatory fibrosis, indicating that PKC*δ* represents a promising target for treating fibrotic diseases like hepatic cirrhosis.

## 1. Introduction

Hepatic fibrosis is a wound healing response to various injuries that may lead to cirrhosis and even hepatocellular carcinoma (HCC) [1]. Chronic liver inflammation usually precedes fibrosis, and it is well known that hepatic macrophages play a critical role in the pathogenesis [2]. Many lines of evidence indicate that hepatic macrophages not only promote the initiation of fibrosis through the recruitment of proinflammatory immune cells and secretion of proinflammatory cytokines in the early stage but also promote the resolution of fibrosis via the secretion of matrix metalloproteinases in the later stages. Several studies have shown that hepatic macrophages are targets for lipopolysaccharide (LPS), and thus, LPS/Toll-like microbial pattern recognition receptors 4 (TLR 4) signaling provides a mechanistic link to ROS signaling. Both LPS and cytokine tumor necrosis factor-α (TNF-α) play key roles in hepatic inflammation through the activation of NF-κB transcription factor [3,4]. 

NF-κB is a central transcription factor involved in the regulation of hepatic stellate cell activation survival and immune response as well as many other important biological processes [5,6,7]. Upon stimulation with an array of stimuli, including a bacterial endotoxin LPS, TNF-α, and oxidative stress, TLR4 receptor induces NF-κB activation through myeloid differentiation factor 88 (MyD88) -dependent signaling, resulting in the production of pro-inflammatory cytokine TNF-α and the pro-inflammatory factor IL-6 [8,9]. Furthermore, NF-κB increases inducible nitric oxide synthases (iNOS) expression and nitric oxide (NO) production [10]. NF-κB is a dimeric complex formed from a multi-subset family, and the canonical p65–p50 complex is the most abundant [11]. Oxidative stress can reportedly induce the activation of the NF-κB signaling pathway through the activation of NF-κB inhibitory factor (IkB) kinase in a PKC*δ*-dependent manner [12]. The mechanism of RelA/p65 transactivation following nuclear translocation and the involvement of PKC*δ* in NF-κB activation in the nucleus remains poorly understood. 

PKC*δ* is generally known as a critical proapoptotic protein in the DNA damage-induced apoptosis; however, it can also function as a survival signal [13,14]. The precise mechanisms by which PKC*δ* controls signaling pathways to protect cells from apoptosis remain to be elucidated. PKC*δ* promotes cell survival via several well-known prosurvival pathways such as NF-κB, serine-threonine kinase Akt, and extracellular regulated kinase (ERK) [15,16]. Another study showed a protective role for PKCδ in response to TNF-α. TNF induced the translocation of PKC*δ* to the nucleus, where it bound to the NF-κB p65 [17]. Novel PKC isoenzymes may be associated with tissue injury and various inflammatory responses. Indeed, the role of PKC*δ* in inflammation and immunity has been confirmed using PKC*δ*-deficient mice [18]. It has been suggested that PKC*δ* modulates the expression of collagen genes and that the upregulation of PKC*δ* is involved in the pathogenesis of fibrotic disorders. These findings suggest that PKC*δ* activation is involved in the progression of inflammatory fibrosis, which is closely connected to hepatic cirrhosis.

Sirtuin-1 (SIRT1) is an NAD(+)-dependent protein deacetylase and functions as a key metabolic sensor in various tissues [19,20]. During chronic inflammation, decreased level of nuclear SIRT1 leads to increased NF-κB RelA/p65 activity and proinflammatory gene expression. It was shown in a *Sirt1* knock-out mouse that the deletion of *SIRT1* in macrophages activates NF-κB activity, thus resulting in the upregulation of proinflammatory genes [21]. Moreover, the significance of SIRT1 in alcoholic liver disease (ALD), nonalcoholic fatty liver disease (NAFLD), and HCC has been widely reported [22,23]. However, the exact regulation of SIRT1 expression levels in liver fibrosis has not been illuminated. In this regard, the level of SIRT1 expression is important in restoring homeostasis during stress responses. Since the level of SIRT1 is regulated through transcriptional processes, the level of expression directly depends on the stability of SIRT1 mRNA. Consequently, the *SIRT1* mRNA half-time is extended, which consequently increases the protein levels.

A causative link between the activation of PKC*δ* and the pathology of inflammatory fibrosis disease remains to be elucidated. In the present study, we explored whether the PKC*δ* signaling in inflammatory fibrosis is involved in the regulation of SIRT1 expression and the regulation of α-Smooth muscle actin (α-SMA) expression through NF-κB. Consequently, we demonstrated that PKC*δ* in the mouse model of CCl_4_-induced hepatic inflammation strongly stimulates the NF-κB inflammatory response. It also demonstrated the involvement of PKC*δ* in the negative regulation of SIRT1 expression in in vitro and in vivo conditions. On the basis of the results, we propose that blocking PKC*δ* activation could be of value to inflammatory fibrosis.

## 2. Results

### 2.1. The Involvement of PKCδ in Liver Fibrosis Induced by CCl_4_

**Figure 1:**
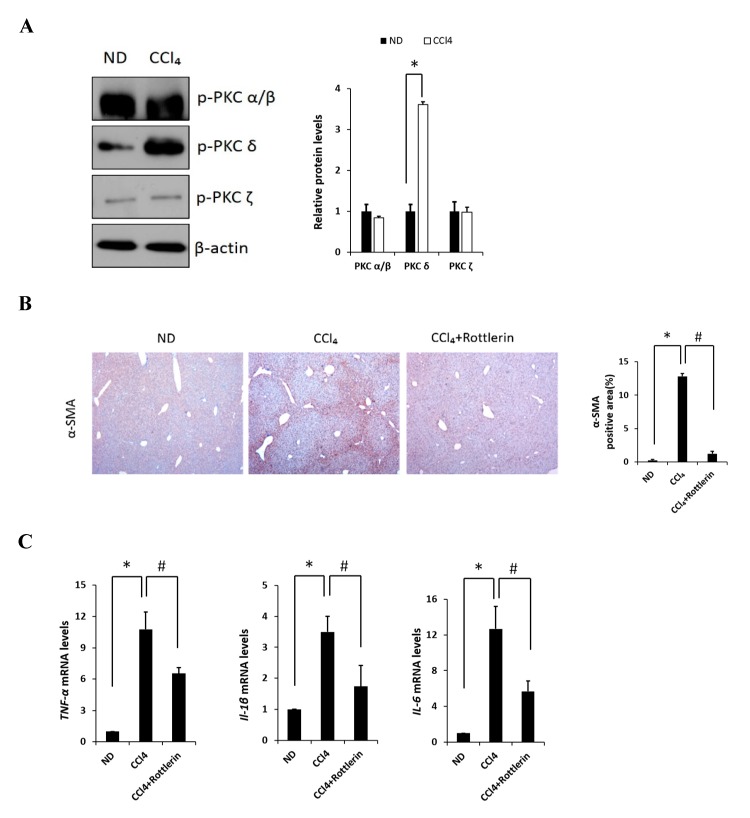
Effects of PKC on carbon tetrachloride (CCl_4_)-induced liver fibrosis in mice. (**A**) Liver phosphorylated PKC isoforms (α/β, δ, and ζ) in ND (Normal diet) and CCl_4_ mice were analyzed by immunoblotting. (**B**) Representative liver tissue sections from ND, CCl_4_, and CCl_4_ + rottlerin mice were stained with α-SMA (original magnification, 100×). (**C**) Hepatic mRNA levels of *TNF-α*, *IL-1β* and *IL-6* were measured by quantitative real-time (qRT)-PCR. Genes were normalized to 18s rRNA as an internal standard, and the data are expressed as fold increase. Data are presented as mean ± SD of three experiments. * *p* < 0.05 compared to the ND group, and # *p* < 0.05 compared to the CCl_4_ group.

Carbon tetrachloride (CCl_4_) is one the most commonly used hepatotoxic agents in experimental animals in the study of liver fibrosis and cirrhosis. CCl_4_ is metabolized by Cytochrome P450 2E1 (CYP2E1) to a trichloromethyl radical, which causes hepatocellular damage through several free radical reactions and lipid peroxidation processes [24]. To investigate the involvement of PKC in CCl_4_-induced acute liver fibrosis, we tested for the different isoforms in the ND (normal diet) and CCl_4_-treated mice (Figure 1A). Among the PKC isoforms tested in this study, hepatic phosphorylated PKC*δ* (p-PKC*δ*) was dramatically increased in the CCl_4_-treated mice when compared to that in the ND mice injected with saline, whereas no significant differences were observed in p-PKC *α*/β or ζ groups. These results indicated that p-PKC*δ* could be specifically involved in CCl_4_-induced liver inflammation. To further confirm whether PKC*δ* activation is implicated in hepatic fibrosis, a specific PKC*δ* inhibitor, rottlerin, was administered to the mice. CCl_4_ administration resulted in increased levels of aspartate transaminase (AST) and alanine transaminase (ALT), both indicative of liver damage, whereas the treatment with rottlerin significantly prevented the increase of these enzymes (Figure S1A). It should be pointed out that the involvement of PKC*δ* in CCl_4_-induced liver injury has never been explored. Immunohistochemistry and immunoblotting analysis showed that the level of α-SMA was dramatically increased in CCl_4_-induced liver tissues. However, the expression of α-SMA was significantly decreased in rottlerin-treated CCl_4_ mice when compared with CCl_4_ mice (Figure 1B). These results were confirmed by immunoblotting analysis (Figure S1B). The mRNA levels of *TNF-*α**, *myd88*, *IL-1β*, *IL-6*, *cox-2*, and *iNOS* were measured in the livers of mice. As shown in Figure 1C and Figure S1C all expression levels were markedly increased in the CCl_4_-treated mice as compared to ND mice (* *p* < 0.05); however, these increased levels were significantly downregulated again by rottlerin treatment. Taken together, these results indicated that the activation of PKC*δ* is specific in CCl_4_-induced liver fibrosis. 

### 2.2. Regulation of NF-κB Signaling by PKCδ in RAW 264.7 Cells

**Figure 2:**
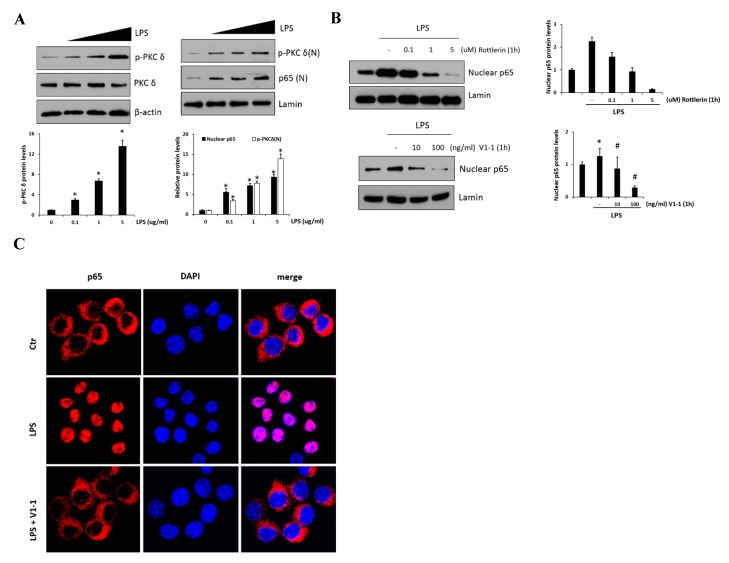
PKC*δ* up-regulates NF-κB translocation in lipopolysaccharide (LPS)-stimulated RAW 264.7 macrophages. (**A**) RAW 264.7 macrophages were treated with or without LPS (0.1, 1, and 5 μg/mL), followed by the measurement of phosphorylation or the expression of PKC*δ* (left panel). The right panel represents nuclear p65. Cells were treated with LPS (0.1, 1, and 5 μg/mL), followed by the measurement of nuclear p65 in lysates. The graphs on the right show the relative amount of the measured protein as fold-increase. * *p* < 0.05 compared to the control group, and # *p* < 0.05 compared to the LPS treatment group. Each value is the mean ± SD of three experiments. (**B**) Cells were pretreated with different concentrations of rottlerin (0.1, 1, or 5 µM, the upper panel) V1-1 (10–100 ng/mL, the lower panel) for 1 h and then incubated with LPS (1 μg/mL); this was followed by the measurement of nuclear p65 and total p65 by immunoblot assay. Lamin was used as a loading control for the nuclear protein. (**C**) Cells were pretreated with or without V1-1 for 1 h, followed by incubation with 1 μg/mL LPS additional and then immunostained with DAPI and anti-p65 Abs. Cells were visualized by fluorescence microscopy (original magnification, 200×).

LPS-mediated TLR4 activation and NF-κB inflammatory signaling are believed to play an essential role during the development of the liver fibrosis. Thus, in vitro, we used RAW 264.7 cells to examine the effect of PKC*δ* regulation in inflammatory signaling on LPS-induced macrophage activation. As shown in Figure 2A, p-PKC*δ* and nuclear p65 induced by LPS (0.1, 1, and 5 μg/mL) were measured by immunoblotting in the cells. The left panel shows the levels of phosphorylated PKC*δ* in response to LPS which were upregulated in a dose-dependent manner. Moreover, we found that the practical modulation of p-PKC*δ* and p65 levels occurred in the nucleus (right panel). To block the signaling in the RAW 264.7 cells treated with 1 μg/mL LPS, we administered the specific PKCδ inhibitors, a peptide V1-1, and rottlerin (Figure 2B). Although rottlerin has been used as a PKC*δ* inhibitor, recent reports have indicated a problem regarding the specificity [25]. Therefore, we used a peptide inhibitor V1-1 that blocked the activation of PKC*δ* more specifically. Pretreatment with rottlerin (0.1–5 µM) dose-dependently downregulated nuclear P65 levels (upper panel). The decrease in nuclear P65 levels was similar in cells treated with PKC*δ* V1-1 (10 and 100 ng/mL) (lower panel). We also investigated whether the nuclear transcription factor p65 was translocated to the nucleus during TLR4 signaling. As shown in Figure 2C, immunofluorescence showed that NF-κB p65 accumulation in the nucleus was strongly induced when the cells were exposed to LPS. However, the translocation of p65 to the nucleus was not observed after pretreatment with V1-1 peptide. All these results indicated that PKC*δ* regulates p65 nuclear translocation in LPS-stimulated RAW 264.7 cells.

### 2.3. PKCδ Negatively Regulates SIRT1 Activation

**Figure 3:**
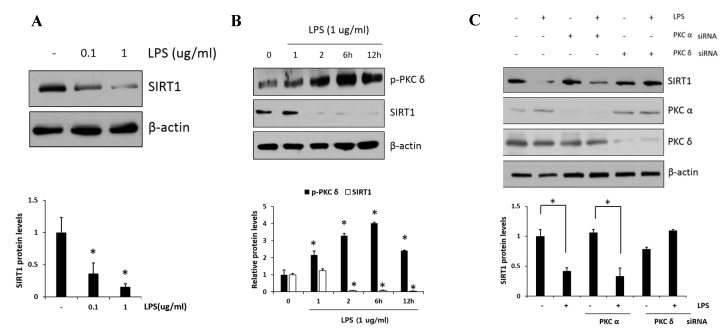
Effect of PKC*δ* on LPS-induced sirtuin-1 (SIRT1) expression in RAW 264.7 macrophages. (**A**) Cells were treated with LPS (0.1 and 1 μg/mL), followed by SIRT1 measurement. (**B**) Cells were treated with LPS (1 μg/mL), followed by the measurement of phosphorylated PKC*δ* and SIRT1 in lysates after 1, 2, 6, and 12 h. (**C**) Cells were transfected with PKCα or PKC*δ* siRNA and after 24 h and were then treated with or without LPS (1 μg/mL), followed by the measurement of SIRT1 with PKCα and PKC*δ*. Values are presented as mean ± SD. * *p* < 0.05 compared with control group.

PKC*δ* has been implicated as an important modulator in the regulation of nuclear p65; however, whether or not PKC*δ* activity is involved in the regulation of SIRT1 expression has remained unclear. First, we examined the SIRT1 expression in response to LPS (0.1 and 1 μg/mL) in RAW 264.7 cells. As shown in Figure 3A, we confirmed that LPS induced dose-dependent decreases in SIRT1 expression. Moreover, the level of p-PKC*δ* induced by the treatment of 1 μg/mL LPS was increased and peaked at 6 h, whereas SIRT1 expression was reversely decreased to a low level within 2 h and maintained the level thereafter (Figure 3B). The effects of siRNA-mediated knockdown regulation of PKC isoform on SIRT1 level were investigated (Figure 3C). In the presence of LPS, the level of SIRT1 in PKC*α* siRNA transfectants was downregulated similarly to that in control cells, whereas the level in PKC*δ* siRNA transfectants was further augmented. These results indicated that PKC*δ* but not PKCα is specifically involved in LPS-induced downregulation of SIRT1. 

### 2.4. Negative Regulation of SIRT1 to the NF-κB Signaling 

**Figure 4:**
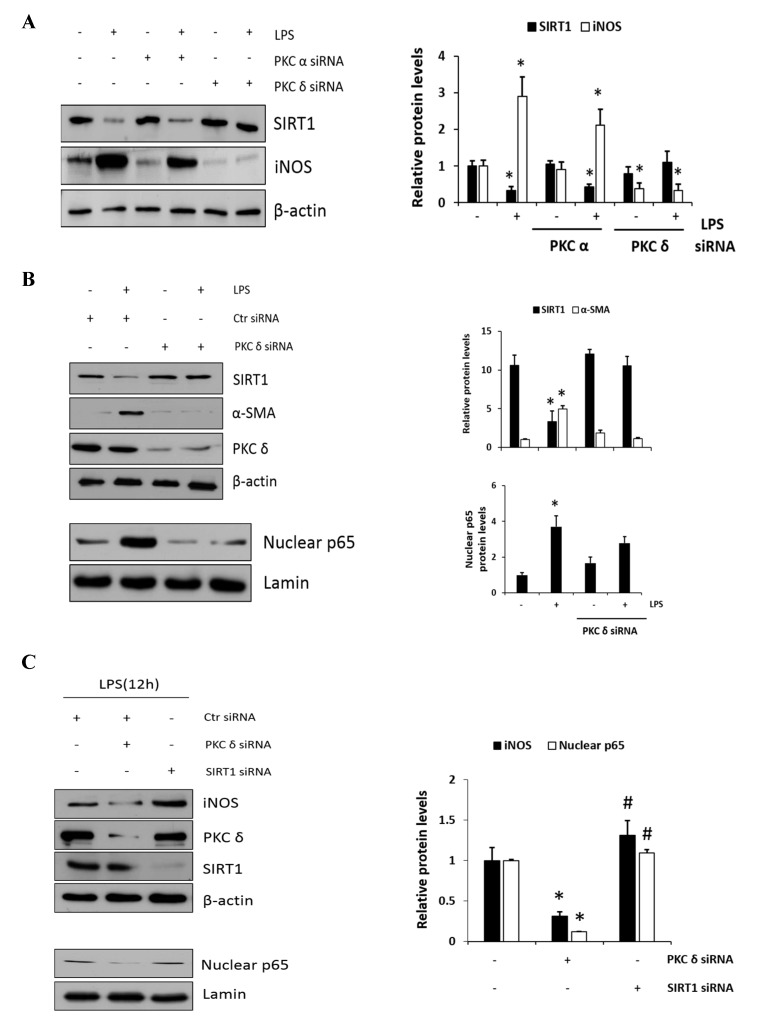

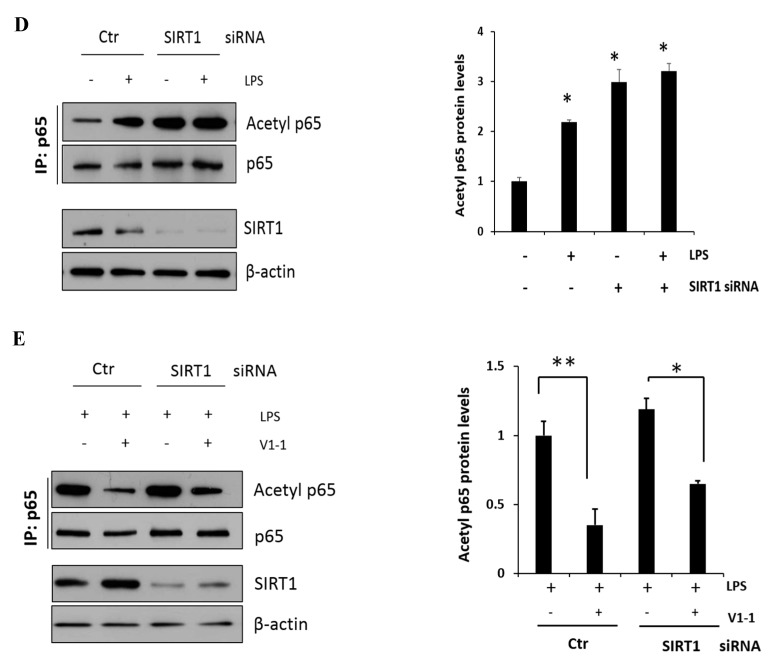
A negative cross reaction between NF-κB and SIRT1 activation in RAW 264.7 macrophages. (**A**) RAW 264.7 cells were transfected with PKCα or PKC*δ* siRNA for 24 h, and then, the cells were either not treated or treated with LPS (1 μg/mL) followed by the measurement of SIRT1 and iNOS. (**B**) Cells were transfected with control or PKC*δ* siRNA, and after 24 h, cells were either not treated or treated with LPS (1 μg/mL), followed by the measurement of SIRT1 and α-SMA. Similarly, the levels of nuclear p65 were also measured with lamin. The relative amounts of the above proteins are presented as fold-increase. The data represent three independent experiments. * *p* < 0.05 compared with control. (**C**) Cells were transfected with PKC*δ* or SIRT1 siRNA after 24 h and then treated with LPS (1 μg/mL) followed by the measurement of iNOS with the transfectant proteins PKC*δ* and SIRT1. The lower panel shows the level of nuclear p65 expression with lamin as the loading control. * *p* < 0.05 compared with control siRNA; # *p* < 0.05 compared with the PKC*δ* siRNA. (**D**) Cells were transfected with SIRT1 siRNA for 24 h, and then, the cells were either not treated or treated with LPS (1 μg/mL) for 1 h. p65 was immunoprecipitated with anti-p65 antibody using whole-cell lysate and then immunoblotted against acetyl-lysine using the same amount of the precipitate. The lower panel confirms the dependency of siRNA transfection on SIRT1 expression. * *p* < 0.05 compared with control. (**E**) Similarly, the effects of PKC*δ* and SIRT1 on the acetyl p65 level in the presence of LPS were shown. The control and SIRT1 siRNA transfected cells were pretreated with or without V1-1 for 1 h, followed by incubation with 1 μg/mL LPS additional. * *p* < 0.05 and ** *p* < 0.01 compared with LPS-induced cells in the absence of V1-1.

To further confirm the involvement of PKC*δ* activity in SIRT1 expression, cells were transfected with either PKC*α* or PKC*δ* siRNAs and were then either not treated or treated with LPS; this was followed by immunoblotting to measure SIRT1 and iNOS expression level. As shown in Figure 4A, the knockdown of PKC*δ* with LPS treatment did not decrease but rather maintained SIRT1 expression, whereas iNOS expression did not increase conversely. However, the knockdown of PKC*α* had much lesser effect on this process like the control siRNA-transfected cells. These results indicated that LPS-induced PKC*δ* activation may play a significant role in SIRT1 and iNOS production. Furthermore, as expected, SIRT1 was decreased in response to LPS, whereas α-SMA was increased (Figure 4B, the upper panel). However, the knockdown of PKC*δ* neither decreased the expression of SIRT nor increased the level of α-SMA. Similarly, the knockdown of PKC*δ* with LPS treatment did not increase the p65 level in the nucleus (Figure 4B, the lower panel). Cells were transfected with PKC*δ* or SIRT1 siRNA, and then, LPS-induced iNOS and nuclear p65 levels were measured (Figure 4C). Results in the upper panel show that PKC*δ* siRNA-transfected cells significantly decreased iNOS, but the SIRT1 transfectants rather augmented as compared to control level of iNOS. Conversely, the levels of nuclear p65 in PKC*δ* and SIRT1 siRNA transfectants were decreased and did not further increase as compared to control siRNA-transfected cells (the lower panel). The levels of acetyl p65 were directly determined using immunoprecipitation followed by immunoblotting analysis (Figure 4D). The level of acetyl p65 in response to LPS was significantly increased in the control siRNA-transfected cells, whereas the level in SIRT1 siRNA-transfected cells was augmented without LPS but had no additional effect in response to LPS. Similarly, the cells were transfected with SIRT1 siRNA followed by immunoprecipitation, and then, LPS-induced acetyl p65 levels were measured in the absence or presence of the PKC*δ* inhibitor V1-1 (Figure 4E). In response to V1-1, the level of acetyl p65 was significantly increased in SIRT1 siRNA cells than in control siRNA cells. Taken together, these results conclusively indicated that PKC*δ* promotes NF-κB signaling but negatively regulates the SIRT1 level simultaneously, and the NF-κB p65 level is negatively regulated by SIRT1 deacetylation activity.

### 2.5. Effects of PKCδ Inhibitor on CCl_4_-Induced Liver Fibrosis in Mice

**Figure 5:**
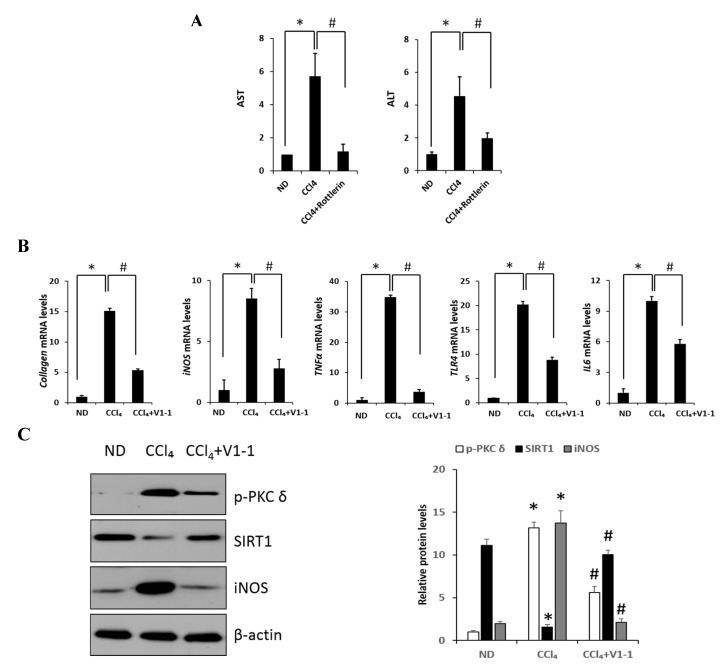

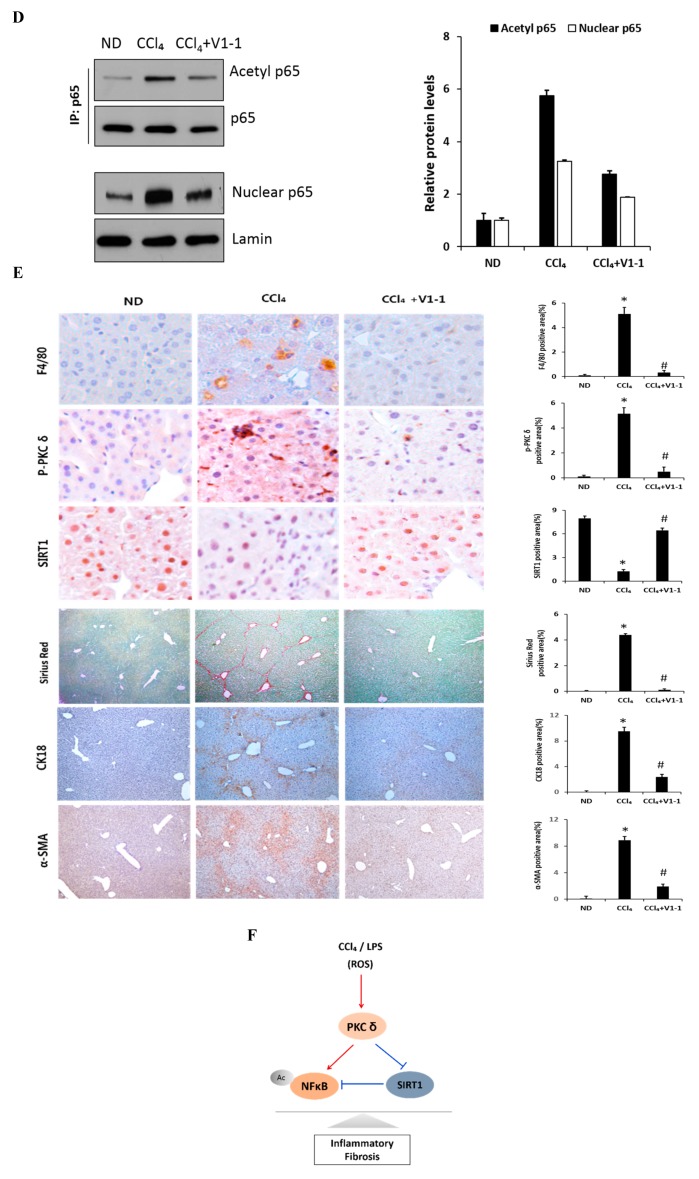
PKC*δ* peptide inhibitor attenuates the progression of CCl_4_-induced liver fibrosis in mice. (**A**) Serum aspartate transaminase (AST) and alanine transaminase (ALT) levels from the control, CCl_4_, and CCl_4_ + V1-1 group mice. (**B**) The mRNA levels of *collagen*, *iNOS*, *TNF-α*, *TLR4*, and *IL-6* in liver tissue homogenates were measured by qRT-PCR. Each value is the mean ± SD of three experiments. (**C**) The expression levels of p-PKC*δ*, SIRT1, and iNOS in the liver tissue were measured by immunoblotting analysis. (**D**) Liver tissue lysates were subjected to immunoprecipitation with anti-p65 antibodies. Immunoblot analysis was then used to detect the levels of acetyl p65 and total p65 in the immunoprecipitated complexes with anti-acetyl-lysine and anti-p65 antibodies, respectively (upper panel). The lower panel represents immunoblot analysis to detect nuclear p65 levels in liver tissue lysates without immunoprecipitation. (**E**) Representative liver tissue sections from ND, CCl_4_, and V1-1 peptide-treated CCl_4_ mice were subjected to immunohistochemical analysis for F4/80, phosphorylated PKC*δ*, and SIRT1 (upper panel group; original magnification, 200×) and Sirius Red, CK18, and α-SMA (lower panel; original magnification, 100×). * *p* < 0.05 compared to the control and # *p* < 0.05 compared to the CCl_4_ group. (**F**) The proposed signaling pathway for liver fibrosis where PKC*δ* upregulates NF-κB signaling but downregulates SIRT1 signaling simultaneously. In addition, SIRT1 directly downregulates NF-κB through deacetylation.(Red arrow: activation and Blue bar: inhibition).

Here, we verified the LPS-induced PKC*δ* signaling and examined whether the inhibition of PKCδ with V1-1 in CCl_4_ mice could be useful for inflammatory fibrosis. Similar to that observed with rottlerin treatment, the administration of V1-1 to the CCl_4_ mice resulted in a significant amelioration (Figure 5A). Moreover, we confirmed that treatment with the PKC*δ* inhibitor V1-1 affectively ameliorated pathological lesions caused by CCl_4_. To explore the levels of proteins involved in the development of liver fibrosis, we performed qRT-PCR analysis of the liver homogenate. The mRNA levels of *TNF-α, TLR4, collagen, IL-6*, and *iNOS* in CCl_4_-treated mice were dramatically increased as compared with those in ND mice, whereas V1-1 treatment significantly suppressed the expression of these genes (Figure 5B). To verify the proteins involved in PKC*δ* signaling, we also performed immunoblotting analyses (Figure 5C). Both p-PKC*δ* and iNOS were significantly upregulated in CCl_4_-induced mice but were suppressed by V1-1 treatment. We also showed that the effect of CCl_4_ on SIRT1 expression was significantly downregulated but was upregulated to the ND level by V1-1 treatment. As expected with the results of LPS-stimulated cells, the levels of SIRT1 expression reversed each other during CCl_4_-induced liver damage. We also confirmed that this translocation process was restored by V1-1 treatment. To confirm the involvement of acetylation in p65 activation, immunoprecipitation analysis with p65 antibody was performed, followed by immunoblotting using the acetyl-lysine antibody. The upper panel in Figure 5D shows an increase in the acetylation of p65 with CCl_4_ treatment but significant suppression of acetylated p65 levels after further treatment with V1-1 peptide inhibitor. Moreover, the levels of nuclear p65 were significantly increased during liver damage with CCl_4_, but treatment with V1-1 resulted in significant recovery. To characterize the phenotype of the macrophages accumulated during liver damage, immunohistochemistry analysis was performed on F4/80, a marker of murine macrophages. The liver histology showed that hepatic macrophage accumulation increased in CCl_4_ mice to a greater extent than in ND mice but was reduced by V1-1 treatment (Figure 5E, upper panel). We also confirmed a reciprocal correlation between p-PKC*δ* expression and SIRT1. The p-PKC*δ* level was significantly increased in CCl_4_ mice, whereas SIRT1 was reversely decreased. Moreover, Sirius Red staining exhibited extensive collagen deposition, indicative fibrosis, but this fibrosis was significantly ameliorated by V1-1 treatment. Similarly, the levels of cytokeratin 18 (CK18) and α-SMA were upregulated in CCl_4_ mice but downregulated in V1-1-treated mice (Figure 5E, lower panel). These results strongly suggested that inhibiting hepatic activity with V1-1 ameliorates fibrosis. In conclusion, we proposed that CCl_4_-induced p-PKC*δ* regulates the NF-κB and SIRT1 pathway in parallel but inversely (Figure 5F). Thus, the NF-κB p65 level is upregulated by PKCδ activation, whereas SIRT1 is downregulated. Furthermore, p65 can be deacetylated by SIRT1, leading to downregulation of NF-κB activity. 

## 3. Discussion

CCl_4_ is well known to cause severe liver injury via ROS elevation, thus leading to oxidative stress, inflammatory response, apoptosis, and tissue necrosis [26,27]. TLR4 is reportedly involved in the CCl_4_-induced hepatic injury, and NF-κB has a key role in the inflammatory response [28]. NF-κB can play a conflicting role in promoting both cell survival and cell death. However, the underlying molecular mechanism that dictates the effect of NF-κB activation on inflammation remains to be elucidated. Conversely, several lines of evidence indicated that PKCδ is involved in the stimulatory effect of NF-κB in hepatic fibrogenesis and steatohepatitis [29]. The role of PKC*δ* in the pathogenesis of hepatic fibrotic disorders, such as hepatic cirrhosis, also remains unknown. Thus, we sought to determine a causative link between the activation of PKC*δ* and the pathology of liver inflammatory disease. Herein, the present study had two major objectives: To determine the involvement of CCl_4_ or LPS in the NF-κB signaling pathway through PKC*δ* and to investigate how PKC*δ* activation is associated with SIRT1 expression. 

The relationship between CCl_4_ and PKCδ in inflammatory fibrosis disease remains completely unknown. In the present study, we have clearly shown that PKC*δ* is activated by CCl_4_ treatment, which is closely linked to the NF-κB inflammatory response signaling. The NF-κB transcription factor triggers the expression of crucial pro-inflammatory genes, including *TNF-α, IL-6,* and *IL-1β* in CCl_4_-treated mice in vivo as well as in LPS-induced liver cells in vitro. Furthermore, iNOS expression and nitric oxide (NO) production are upregulated in response to LPS, appearing to be mediated by NF-κB activation [30]. Conversely, the inhibition of PKC*δ* with V1-1 peptide led to a dramatic reduction in the NF-κB p65 activity. Although the activation mechanism is not clear, it is known that the activated PKC*δ* by various stimuli is targeted to the nucleus and phosphorylates several nuclear targets, including p65 [31]. In this regard, we confirmed an increase in activated PKC*δ* by LPS in the nucleus (Figure 2A). Notably, PKC*δ* activation would be a prerequisite for its nuclear targeting and the subsequent binding to p65, and the V1-1 PKC*δ* inhibitor is used as a specific blocker for cytoplasmic-to-nuclear transfer. Consequently, CCl_4_ activates PKC*δ* and elicits an inflammatory fibrosis response through the NF-κB signaling pathway. Therefore, we propose that blocking PKC*δ* activation could be valuable in managing fibrotic diseases, such as liver cirrhosis; nevertheless, there are many unanswered questions that need further investigation.

The second issue to be addressed is the mechanism by which PKC*δ* regulates SIRT1 expression. In earlier studies, SIRT1 has been proven to be associated with ALD or NAFLD [32]. SIRT1 plays important roles in regulating lipid metabolism, hepatic oxidative stress, and inflammation in the liver [33,34]. Moreover, several studies have revealed the SIRT1 represses inflammatory signaling in hepatic macrophages and RAW 264.7 cells [35]. Furthermore, we show that PKCδ co-localizes with SIRT1 in RAW 264.7 cell (Figure S2). However, little research has focused on the role of SIRT1 in liver fibrosis. In addition, a decrease in the level of SIRT1 was reported in a fibrosis model [36,37]; however, the regulatory pathway remains largely unknown.

Several pathways are known for the regulation of SIRT1 expression. SIRT1 levels are transcriptionally regulated by nutrient availability, and the expression of *SIRT1* gene is inhibited by Carbohydrate response element binding protein (ChREBP) in obesity or diabetes [38]. In addition, recent studies have suggested that miRNAs at post-transcriptional levels are responsible for decreasing SIRT1 level [39]. In this study, we demonstrated that SIRT1 expression was PKC*δ* dependent, whereas treatment with PKC*δ* peptide inhibitor dramatically upregulated SIRT1 expression. In other words, SIRT1 expression was inversely controlled by PKC*δ* activities and was thought to contribute to the reduction of pro-inflammatory cytokines. Although we demonstrated that PKC*δ* activation is negatively associated with SIRT1 expression, no direct linkage has been found between them. In addition to the importance of the PKC*δ*–NF-κB signaling axis and the PKC*δ*–SIRT1 axis, we cannot overlook the contribution of SIRT1 to this NF-κB signaling function. To investigate the importance of SIRT1 for NF-κB-dependent transcription, we used two complementary approaches: A pharmacological approach using the V1-1-term PKC*δ* peptide and a silencing PKC*δ* using siRNA transfection. Thus, we confirmed that the inhibition of PKC*δ* in CCl_4_ mice or LPS-treated cells significantly reduced the levels of acetyl p65 and p65 in the nucleus. In addition, we have clearly demonstrated the recovery of SIRT1 through the V1-1 or PKC*δ* siRNA (Figure 3C and Figure 5C). In particular, the level of p65-dependent gene expression and the presence of p65 in the nucleus appeared to be closely related to SIRT1 activity. PKC*δ*-mediated p65 is acetylated in the nucleus, which is important to maintain its nuclear localization and the consequent NF-κB transcriptional activity [40]. The deacetylation of RelA/p65 decreases the NF-κB transcription activity, thus reducing the production of proinflammatory cytokine genes. Therefore, it seemed that the upregulation of SIRT1 by PKC*δ* inhibition mainly downregulated NF-κB transcription through the deacetylation of p65 and contributed to the export of nuclear p65–NF-κB to the cytoplasm.

Here, we primarily focused our effort on determining the role of PKC*δ* activation in inflammatory fibrosis. Consequently, we have uncovered several novel characteristics of PKC*δ* in mouse model experiments. First, we noted that the CCl_4_-induced NF-κB signal pathway was mediated by PKC*δ* activation. The induction of *collagen* and α-SMA are considered as markers of liver fibrosis. The expression of α-SMA is upregulated in CCl_4_-treated mouse liver tissues [41,42]. Contrary to the mechanism observed with α-SMA stimulation, a potential regulatory mechanism by which PKC*δ* affects profibrogenic gene expression in response to CCl_4_ stimulation has never been explored. Second, although it is unclear whether directly or indirectly, PKC*δ* activation resulted in the negative regulation of SIRT1. Thus, our data demonstrated for the first time that PKC*δ* activation by CCl_4_ affects the expression of α-SMA via NF-κB signaling and is also involved in negatively regulating SIRT1 expression, suggesting a novel role of PKC*δ* in inflammatory fibrosis. Finally, the other exciting finding from the present study was that hepatic inflammatory fibrosis was significantly decreased by the blockage of the PKC*δ* pathway. We have previously shown that α-SMA was significantly increased in the methionine–choline-deficient (MCD) diet mice and that the inhibition of PKC*δ* ameliorated hepatic steatosis, suggesting PKC*δ* participation in the progression of non-alcoholic steatohepatitis (NASH) [29]. In this study, we demonstrated that NF-κB-dependent proinflammatory genes increased in CCl_4_ mice, whereas SIRT1 expression did the opposite. Interestingly, the inhibition of PKC*δ* by rottlerin or V1-1 led to complete reversal and, hence, dramatically recovered the inflammatory fibrosis. Moreover, the overall improvement in many inflammation-related genes, such as *TNF-α* and *iNOS*, and fibrosis, such as *collagen* and α-SMA, is observed in parallel with increased SIRT1 expression following PKC*δ* inhibition in CCl_4_ mice. Thus, it was clear that the NF-κB-dependent hepatic inflammation can be blocked primarily by the inhibition of PKC*δ* with the upregulation of SIRT1 expression.

## 4. Materials and Methods

### 4.1. Animal Models

C57BL/6J male mice were purchased from Hyochang Science (Daegu, Korea). All mice were conditioned to proper temperature (20–24 °C) and proper humidity (45–55%) for a week. The mice were randomly divided into three groups: Group I (Normal diet group), group II (CCl_4_ group), group III (CCl_4_ + rottlerin group), and group IV (CCl_4_ + V1-1 group). The ND mice were administered daily with sterile saline orally (*n* = 6). In the CCl_4_ model group, liver fibrosis was induced in the mice by intraperitoneally injecting 100 mg/kg of CCl_4_ in olive oil solution three times/week for four weeks (*n* = 8). In the CCl_4_ model + rottlerin or V1-1 group (III, IV), the mice received the same dose of CCl_4_ also treated with rottlerin (Calbiochem, San Diego, CA, USA) or V1-1 (Peptron, Seoul, Korea) for four weeks (*n* = 8). The animal experimental procedures were performed in accordance with approved animal protocols and guidelines established by the Animal Care Committee of Kyungpook National University (No. KNU 2016-42).

### 4.2. Cell Culture and Transfection

The murine macrophage cell line [RAW 264.7; American type culture collection (ATCC), Manassas, VA, USA] was maintained in DMEM supplemented with 10% FBS, 100 μg/L streptomycin, and 100 IU/mL penicillin at 37 °C in a 5% CO_2_ atmosphere. PKCα and PKCδ siRNA (Santa Cruz, CA, USA) transient transfection was performed using Lipofectamine RNAiMAX (Invitrogen, Waltham, MA, USA). Cells were homogenized in NP40 buffer, and the immunoblot procedure was applied. The primary antibodies used were PKCα and PKCδ (Cell Signaling; Danvers, MA, USA) for detecting proteins.

### 4.3. Preparation of Whole-Cell and Nuclear Extracts

RAW 264.7 cells were seeded at 1 × 10^5^ cells/well on well plates and were treated with LPS (Sigma-Aldrich, St Louis, MO, USA) and LPS with V1-1. After treatment, the cells were collected by centrifugation and washed twice with ice-cold PBS. Cytosolic and nuclear proteins were extracted using the NE-PER^®^ Nuclear and cytoplasmic Extraction Reagents (Pierce Biotechnology, Inc, Rockford, IL, USA).

### 4.4. Immunoblot Analysis

Liver tissues and RAW 264.7 cells were homogenized in NP40 buffer containing 50 mM HEPES pH 7.4, 150 mM NaCl, 1% NP40, 1 mM EDTA, and protease inhibitor cocktail tablets (Roche, Mannheim, Germany). After electroblotting, the proteins were separated onto nitrocellulose membranes (GE Healthcare Life science), the membranes were blocked with 5% w/v skim milk in TBST (50-mM Tris pH 8.0, 150-mM NaCl, and 0.1% Tween-20 (*v*/*v*)) for 1 h. The membranes were immunoblotted with antibodies against α-SMA (Sigma-Aldrich, St Louis, MO, USA), acetyl-lysine (Merck Millipore KGaA, Darmstadt, Germany), p-PKCα, p-PKC*δ,* p-PKC ζ, P65, SIRT1, iNOS (Cell signaling; Danvers, MA, USA), PKCα, PKC*δ*, and β-actin (Santa Cruz, CA, USA). β-actin was used as the loading control. Primary antibodies were detected using horseradish peroxidase (HRP)-conjugated secondary antibodies. Membranes were washed in TBST and then probed with the appropriate horseradish peroxidase-coupled secondary antibody (Cell Signaling).

### 4.5. Immunoprecipitation

Protein extracted from the liver tissue or RAW 264.7 cells was incubated with antibody against p65 and Dynabeads™ Protein A IP kit (Thermo Fisher Scientific, Waltham, MA, USA). The protein was extracted from immune complexes precipitated with protein A by boiling with 2× sample buffer. Protein samples were separated by SDS-PAGE and then electro-transferred to a membrane. After IP, immunoblot analysis was performed with antibodies against acetyl-lysine (Millipore, Billerica, MA, USA) and p65.

### 4.6. Immunofluorescence Staining

RAW 264.7 cells were fixed with 4% paraformaldehyde in TBST for 30 min, washed with TBST, and permeabilized 0.1% Triton X-100 in TBS-T for 30 min. Cells were blocked with 3% bovine serum albumin (BSA; Sigma-Aldrich) for 1 h at room temperature. Subsequently, the sections were incubated overnight at 4 °C with primary antibodies against p65 and in TBST, which were supplemented with 3% BSA. The sections were then washed thoroughly in TBST, followed by 1 h incubation in the dark with Alexa Fluor 555-conjugated secondary antibody (Thermo Fisher Scientific) at a dilution of 1:100 in TBST supplemented with 3% BSA. The nuclei were stained with DAPI (Invitrogen), and cells were examined under a Zeiss LSM7 PASCAL confocal microscope (Gottingen, Germany). 

### 4.7. Immunohistochemistry

The livers were extracted from mice, and the slide sections were stained with hematoxylin–eosin (H&E) stain for histological analysis. Other slide sections from the paraffin block of liver tissue were stained with primary antibodies for protein localization. Sample sections were reacted with the appropriate secondary antibodies. The immunohistochemical reactions were visualized using 3,3-diaminobenzidine (DAB) reagent. Image analysis was performed using Image J version 1.52a software.

### 4.8. Real-Time PCR

Total RNA was extracted from the liver tissues using TRIzol reagent (Invitrogen). Reverse transcription was performed using SuperScript III RNase H (Invitrogen). Quantitative RT-PCR was performed using SYBR Green I (Takara, Kyoto, Japan). The results were normalized gene expression of 18s rRNA in all experiments. Table S1 lists the primers used for RT-PCR analysis.

### 4.9. Statistical Analysis

All data are expressed as mean ± SD. Statistical analysis was performed using nonparametric Mann–Whitney *t*-tests. For all experiments, *n* = 6–8 animals per group. Significance was established using Mann–Whitney tests, with significance being considered at * *p* and # *p* < 0.05 or ** *p* < 0.01, corrected for multiple comparisons.

## 5. Conclusions

We demonstrated for the first time that in a CCl_4_-treated mouse fibrosis model, PKC*δ* is a key player in modulating NF-κB via SIRT1 signaling. Our data suggested that PKC*δ* activity is involved not only in the activation of NF-κB that leads to the development of hepatic inflammation but also in reversing the suppression of SIRT1 expression in in vitro and in vivo conditions. Notably, the inactivation of PKC*δ* dramatically suppressed the inflammatory fibrosis, thus indicating a positive correlation with the hepatic cirrhosis. Therefore, although the detailed regulation mechanism needs to be further investigated, the antagonistic role of the PKC*δ* inhibitor may open new possibilities in controlling fibrotic diseases.

## Supplementary Materials

The supplementary materials are available online at https://www.mdpi.com/1422-0067/20/18/4607/s1.
