# Supplementary material for: PKCδ Mediates NF-κB Inflammatory Response and Downregulates SIRT1 Expression in Liver Fibrosis
Source: Int J Mol Sci. 2019 Sep 17;20(18):4607. doi: 10.3390/ijms20184607 (PMC6770793; doi:10.3390/ijms20184607)
Supplement: Supplementary file 1 [file ijms-20-04607-s001.pdf]

Supplementary Materials

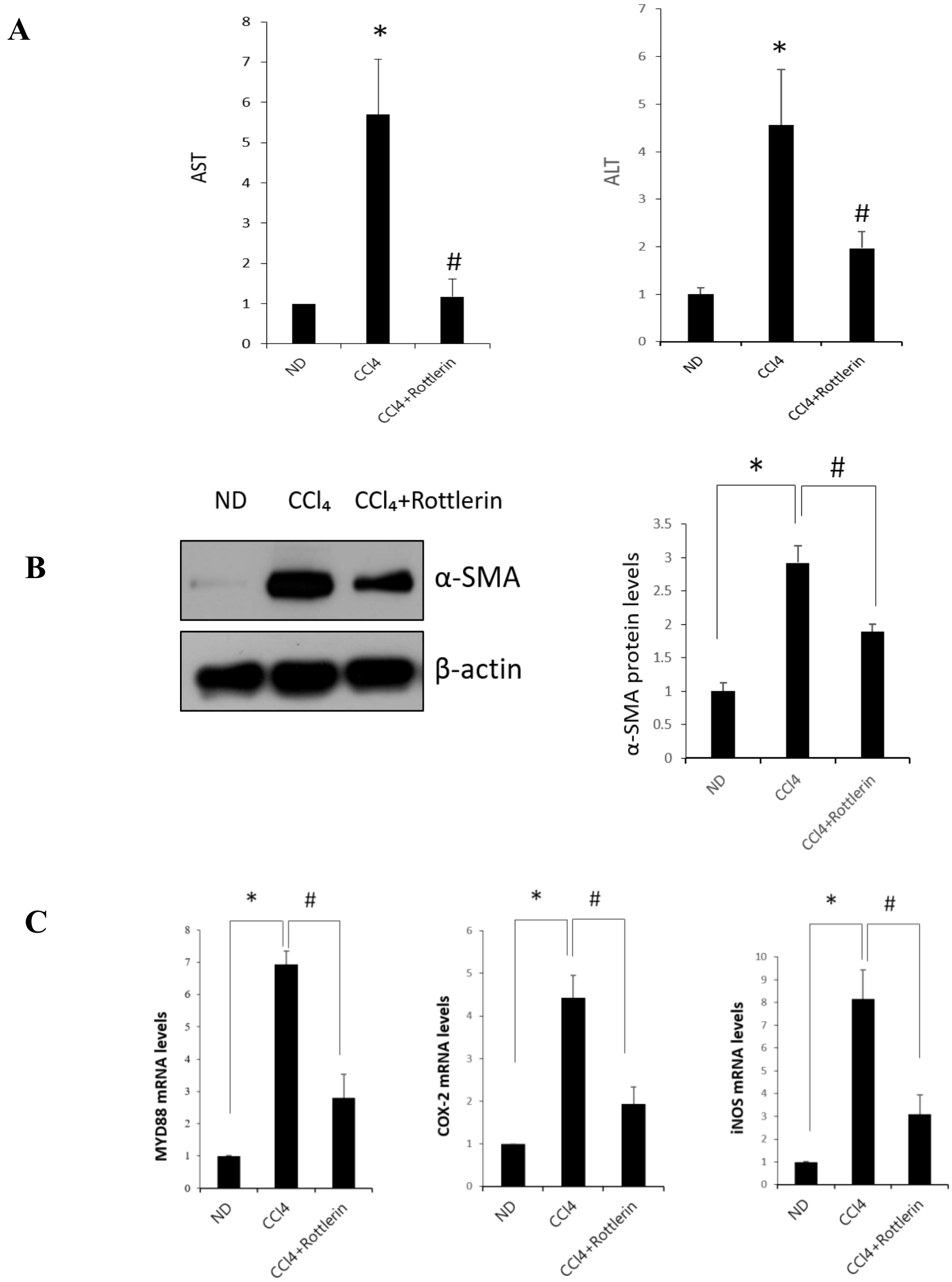

Figure. S1. Effects of rottlerin on carbon tetrachloride (CCl_4)-induced liver fibrosis in mice. (A) Serum AST and ALT levels were measured in CCl4 mice with and without rottlerin treatment. (B) The level of α-SMA in CCl_4 mice after rottlerin treatment was analyzed by immunoblotting. (C) Hepatic mRNA levels of *myd88*, *COX-2*, and NF-*κ*B-mediated the inflammatory cytokine *iNOS* were measured by qRT-PCR.

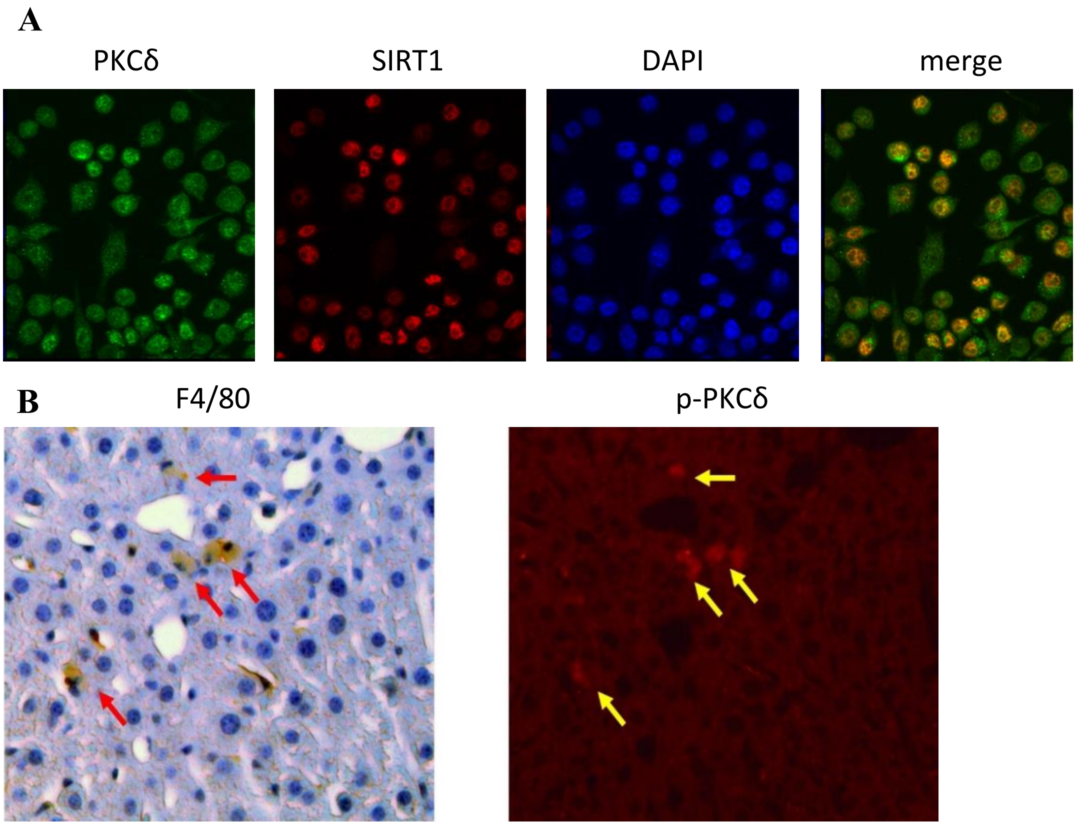

Figure. S2. localization of PKCδ. (A)Colocalization of PKCδ and SIRT1in Raw 264.7 cells. Double Immunofluorescence staining for PKCδ (Green) and SIRT1 (Red) were assessed for colocalization by confocal microscopy (Original magnification, 200×). (B) Colocalization of F4/80 and p-PKCδ in hepatic macrophages in CCl4 model. Double immunohistochemical staining for F4/80(DAB indicated by red arrows) and p-PKCδ (red fluorescence indicated by yellow arrows) were assessed for colocalization by confocal microscopy (Original magnification, 200×).

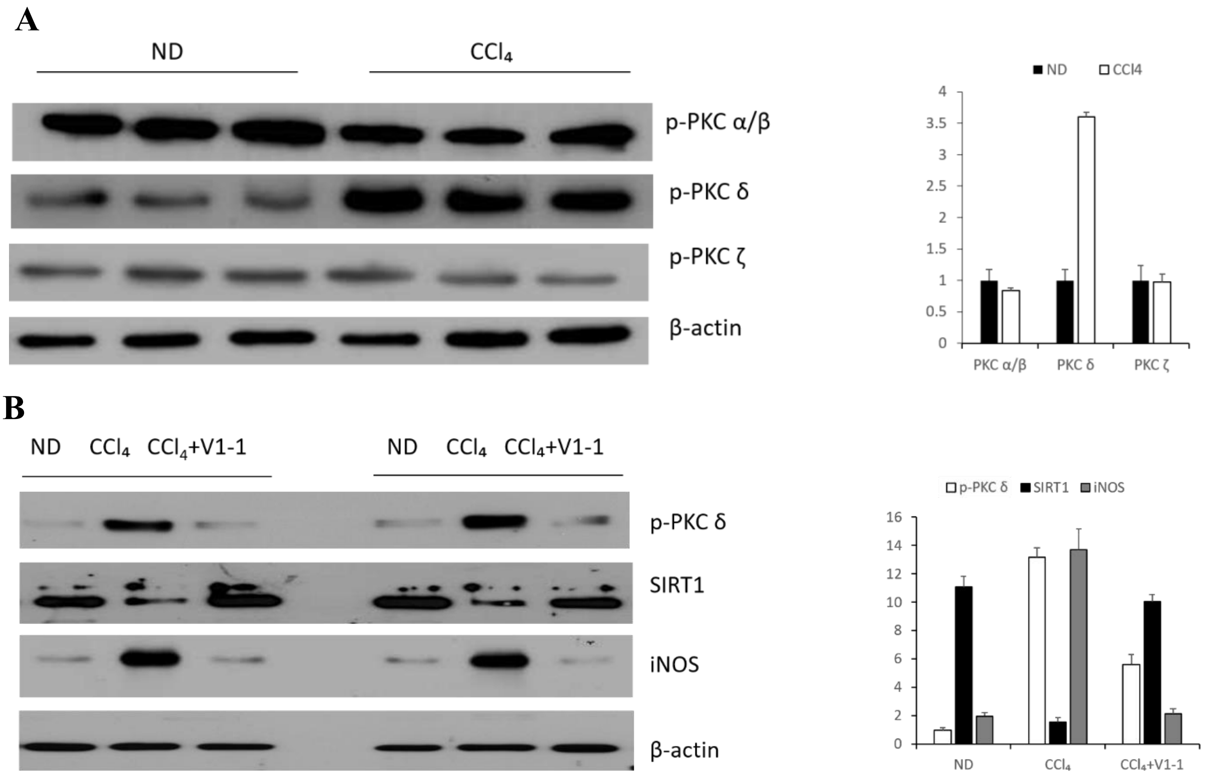

Figure. S3. Western blot assays (representative blots). (A) Fig 1A and (B) Fig 5C

Table S1. Primers used for real-time PCR

**Primer Name**	**Sequences**
18S	(F)5'-GTA ACC CGT TGA ACC CCA TT-3'
	(R)5'-CCA TCC AAT CGG TAG TAG CG-3'
GAPDH	(F)5'-TGG TAT CGT GGA AGG ACT CA
	(R)5'-AGT GGG TGT CGC TGT TGA AG
TNFα	(F)5'-CAC CAC CAT CAA GGA CTC AA-3'
	(R)5'-AGG CAA CCT GAC CAC TCT CC-3'
Myd88	(F)5'-AGA ACA GAC AGA CTA TCG GCT-3'
	(R)5'-CGG CGA CAC CTT TTC TCA AT-3'
COX-2	(F)5'-ATT CCT GGC GTT ACC TTG-3'
	(R)5'-CTG TAT TCC GTC TCC TTG GTT-3'
IL-1β	(F) 5'-GCAACTGTTCCTGAACTCAACT-3'
	(R) 5'-ATCTTTTGGGGTCCGTCAACT-3'
IL-6	(F)5'-GAC AAC TTT GGC ATT GTG G-3'
	(R)5'-ATG CAG GGA TGA TGT TCT G-3'
TLR4	(F)5'-ACC TCT GCC TTC ACT ACA GA-3'
	(R)5'-AGG GAC TTC TCA ACC TTC TC-3'
iNOS	(F)5'-CTG CAG CAC TTG GAT CAG GAA CCT G-3'
	(R)5'-GGG AGT AGC CTG TGT GCA CCT GGA A-3'

collagen	(F) 5'- GAC ATC CCT GAA GTC AGC TGC-3'
	(R) 5'-TCC CTT GGG TCC CTC GAC-3'
SIRT1	(F)5’-TTG TGA AGC TGT TCG TGG AG-3’
	(R) 5’-GGC GTG GAG GTT TTT CAG TA-3’
